# Breast cancer among Danish women occupationally exposed to diesel exhaust and polycyclic aromatic hydrocarbons, 1964–2016

**DOI:** 10.5271/sjweh.3923

**Published:** 2021-03-01

**Authors:** Julie Elbæk Pedersen, Katrine Strandberg-Larsen, Michael Andersson, Johnni Hansen

**Affiliations:** Danish Cancer Society Research Center, Copenhagen, Denmark; Department of Public Health, University of Copenhagen, Copenhagen, Denmark; Department of Oncology, Rigshospitalet, Copenhagen, Denmark

**Keywords:** breast cancer subtype, Denmark, exposure, female worker, full job history, JEM, job exposure matrix, occupational exposure, occupational risk factor, PAH

## Abstract

**Objective:**

The aim of this study was to explore the association between occupational exposure to diesel exhaust and polycyclic aromatic hydrocarbons (PAH), respectively, and breast cancer subtypes.

**Methods:**

The study included 38 375 women <70 years with incident breast cancer, identified in the Danish Cancer Registry, and 5 breast cancer-free controls per case who were randomly selected from the Danish Civil Registration System and matched on year of birth. Full employment history was obtained for all study subjects from a nationwide pension fund, and exposure to diesel exhaust and PAH was assessed using a job exposure matrix. Conditional logistic regression was used for estimation of odds ratios (OR) with adjustment for reproductive factors and socioeconomic status.

**Results:**

No noteworthy associations were observed for overall breast cancer in women exposed to diesel exhaust. However, diesel exhaust modestly elevated the risk of estrogen receptor negative breast tumors before age 50 [OR 1.26, 95% confidence interval (CI) 1.09–1.46]. Duration– and dose–response relationships were further observed for this subtype in this age group. No notable risk patterns were generally observed for PAH exposure.

**Conclusion:**

Occupational exposure to diesel exhaust may increase the risk of early-onset estrogen receptor negative breast tumors in women. Future studies exploring this association are warranted.

Breast cancer has the highest incidence rate of all cancers in women and it has been increasing over the last half of the 20^th^ century (1). Further, breast cancer is a heterogeneous disease, and the various subtypes are often characterized by the presence or absence of particular biomarkers, ie, hormonal and growth receptors (2, 3).

Acknowledged breast cancer risk factors include early age at menarche, late age at menopause, advanced age at childbearing and nulliparity, use of oral contraceptives, and hormone replacement therapy (HTR) (4). In addition, some lifestyle factors such as obesity, alcohol consumption, and physical inactivity as well as greater breast density and genetics are also shown to increase the risk (5, 6). Most risk factors have been observed to be positively associated with different breast cancer subtypes in both young and older women (3, 4). Nonetheless, the etiology of breast cancer is not completely understood and acknowledged risk factors cannot explain all of the increase in incidence rates (7).

More recently, increasing levels of hormone-mimicking chemicals in the environment, including diesel exhaust and polycyclic aromatic hydrocarbons (PAH), have been suggested to play a role in breast cancer development (8) (9). Compared with the general population, certain types of workers especially encounter higher levels of these air pollutants from various work-related sources. Diesel exhaust consists of a mixture of chemical components, including gases (eg, carbon monoxide and nitrogen oxides), particulates comprising metals, sulfates, organic and inorganic carbon, and aromatic hydrocarbons such as benzene and PAH (8). Further, PAH also originate from other sources of incomplete combustion of organic material (9).

The International Agency for Research on Cancer (IARC) has classifed both diesel exhaust and specific PAH as carcinogenic to humans (group 1), but this classification was primarily based on lung cancer (8) (9). Moreover, chemical components of diesel exhaust, including PAH, are lipophilic, have estrogenic properties, and are stored in mammary tissue. Thus, it is plausible that these agents may as well be linked to carcinogenesis in breast cells (10). In addition, the effect of carcinogenic exposures on breast cancer risk among women have been suggested to be higher during particular windows of susceptibility early in life, ie, prior to and during pregnancy (11). Further, the effect may also be higher in nulliparous women as breast tissue in these women is less differentiated (12, 13) and thereby perceived as more vulnerable (14).

Nevertheless, only a limited number of incidence studies have examined the effect of these occupational exposures on the risk of breast cancer among women (15–22). Moreover, breast cancer risk in possibly susceptible subgroups and the risk of hormonal subtypes have not been evaluated in different age groups of women in previous explorations.

Through the use of lifetime information on employment history and a job exposure matrix (JEM), we conducted a nationwide registry-based case–control study exploring the association between occupational exposure to diesel exhaust and PAH, respectively, and the risk of subtypes of breast cancer in different groups of Danish women.

## Methods

### Case and control selection

Cases were initially identified in the national Danish Cancer Registry. Since 1943, this registry has systematically registered all cancers in Denmark and holds individual-based information on the date of diagnosis and details of pathological characteristics. Until 1978, the International Classification of Disease revision 7 (ICD-7) was used to categorize breast cancer. From 1978 to 2003, a converted version of ICD-10 was used and subsequently revision 10 (ICD-10) (23). Employment information was available from 1964 (see next section), and cases with full employment history, ie, women who were born after 1946 and thereby 18 years old in 1964, were included in the study. As a result, cases were ≤70 years old when diagnosed in the study period ending in 2016.

The Danish Breast Cancer Group (DBCG) database was founded in 1975 and has obtained comprehensive information on diagnostics, treatment and control since 1978 (24). We retrieved additional information on subtypes of breast cancer from DBCG covering the period 1978–2015, which included data on estrogen receptor (ER) status.

Demographic information on all residents in Denmark has been registered systematically in the Danish Civil Registration System since 1968 (25). Five controls matched on year of birth were chosen for each case at random using this registry (the incidence-density sampling approach). Controls were required to be alive and free of breast cancer at the date of diagnosis, and controls as well as cases were required to be born in Denmark in order to ensure complete information on work history. The Danish Cancer Registry, DBCG, and the Danish Civil Registration System all entail a unique 10-digit personal identification number (PIN), which serves as a unique key to all public administration of Danish residents, including healthcare, and all relevant information in these registries was linked using this PIN. As the study was registry-based, no ethical approval was required.

### Occupational history

The Danish Supplementary Pension Fund Register (ATP) was established in 1964 and has held compulsory membership for all wage earners working ≥9 hours/week. All jobs are registered with information on the unique PIN of the employee, date of start and end of each employment, company name, and a unique 8-digit company number for tax purposes. All information is kept even if a company closes or employees emigrate or die (26). Statistics Denmark has classified companies into a five-digit branch/industry code (in Danish: Danmarks Statistisk Erhvervsgrupperingskode, DSE) (27) according to an extended version of the International Standard Industrial Classification of all Economic Activities (ISIC) (26, 28). The PIN was also used to link the occupational history from ATP, and registration in this registry, which was an indicator of labor force participation, was a prerequisite for being eligible for the study.

### Exposure assessment

To translate the industrial employments into exposure to diesel exhaust and PAH, respectively, a Danish JEM (NOCCA-DANJEM) was used, which includes metrics of the probability of job-specific exposure (P) and mean intensity level of exposure (L) for each relevant agent of exposure and occupational group. Further, this information is available for four periods of time (1945–1959, 1960–1974, 1975–1984 and 1985–1995).

The JEM is based on the Nordic Occupational Cancer Study job exposure matrix (NOCCA-JEM) (29), and as exposed jobs in the NOCCA-JEM are founded on the Nordic Classification of Occupations (NYK), the construction of the NOCCA-DANJEM had involved a development of a crosswalk between Nordic NYK and Danish DSE codes, which was conducted by a Danish occupational expert. As specific DSE77 codes corresponded to several NYK codes with various exposure estimates, the development of this crossroad had involved an expert evaluation of the average exposure in these Danish industries representing various jobs in which both men and women are employed.

In order to improve specificity, we categorized employment with a probability of exposure ≤20% and women employed less than one year as unexposed. As the JEM did not entail measurements of exposure after 1995, metrics of exposure probability and level for this period were assumed to reflect those in the most recent era (1985–1995).

### Covariates

In the supplementary material (www.sjweh.fi/show_abstract.php?abstract_id=3923), figure S1 provides a directed acyclic graph (DAG) with an overview of the presumed causal interplay of risk factors for breast cancer, and obtainment of information on potential confounders was partly based on this. As a result, we included information from the Danish Civil Registration System on job title reflecting socioeconomic status (SES) and reproductive factors were included. Information on last known job title was self-reported and was initially attained from annual tax returns or official change of address forms. We grouped this information according to SES using the Danish Institute of Social Sciences’ definition: group 1 (highest status) included academics, group 2 included middle education, group 3 included shorter education, group 4 included skilled workers, and group 5 included unskilled workers (30). Further, group 6 consisted of women with missing information on job title, which comprised a relatively large proportion of especially the younger women included in the study (approximately 30%). Information on reproductive factors, including parity (0, 1–2, ≥3 children) and the exact age at first live birth (<25, 25–29, 30–34 and ≥35 years), was available. In addition, possible confounding by work-related physical activity was assessed using the NOCCA-DANJEM and categorized according to “ever” versus “never” having worked in a job with “heavy or rather heavy physical work”.

### Statistical analysis

We used conditional logistic regression for matched data sets to estimate odds ratios (OR) with corresponding 95% confidence intervals (CI), and all analyses were thereby conditioned on the matching variable, ie, year of birth. The full adjusted models further included age at first live birth, parity and work-related physical activity. Due to the relatively large proportion of women who had missing data on SES, we conducted a subsequent full case analysis including women with no missing data on this variable, which yielded analogous results (only results for the overall findings are shown). All analyses were stratified according to the women’s age at the index date (<50, ≥50 years old), approximating menopausal status.

We used different dimensions of exposure, including duration of exposure as well as cumulative exposure. Duration of exposure was calculated by summing the years of employment in all exposed calendar periods across all industries (1–9, 10–20, >20 years). Further, cumulative exposure was the product of the proportion exposed, intensity level and years worked in each exposed time period, which was summed over all exposed jobs in a woman’s occupational history. The categorization of cumulative exposure was based on the percentiles among the controls (>0–25, >25–50, >50–75, >75). Trend tests were conducted to explore duration- and dose–response relationships by using ordinal scores.

Analyses exploring the impact of lag time (the years between initial exposure and the index date) on the main results (>1–9, 10–20 and >20 years) as well as timing of exposure among parous women (before versus after first live birth) were conducted as well. In addition, the same analyses were conducted with a further stratification by ER status. We also conducted a stratified analysis exploring the effect of parous status on the risk of overall breast cancer in the two age groups. Lastly, as the population of women in the study were ≤70 years of age, the lag time for women exposed late in their work life would have been too short for potential breast tumors to develop. Hence, we conducted a sensitivity analysis excluding women exposed after age 50.

All analyses were performed with Stata statistical software v 14.2 (StataCorp, College Station, TX, USA).

## Results

A consequence of restricting the study population to women aged ≤70 years in 2016 was the relatively high proportion of breast cancer cases diagnosed before age 50. The distribution of known major breast cancer risk factors in both age groups was consistent with current knowledge, ie, cases were generally more likely than controls to have a higher SES, lower parity, and higher age at first full-term birth, although a slightly higher proportion had never been employed in work with heavy physical requirements (table 1). Regarding ER status, a relatively larger proportion of cases in both age groups were diagnosed with ER+ tumors and thus the distribution of hormonal cancer subtypes followed an expected pattern as well. Approximately 7% and 6% of the study population of women had been occupationally exposed to diesel exhaust and PAH, respectively, from working in industries entailing these exposures. Women exposed to diesel exhaust had most often been employed in construction as well as different transportation industries entailing this exposure whereas women exposed to PAH most often had been employed in industries involving work with metal and machinery (see supplementary table 1 for a complete overview of the proportion of exposed women employed in industries with diesel exhaust and PAH, respectively).

**Table 1:** Characteristics of included cases and matched controls among the population of Danish women included in the study (N=230 250) by age at diagnosis. [SD=standard deviation.]

	<50 years				≥50 years			
	
	Cases (N=17 332) %	Mean (SD)	Controls (N= 86 660) %	Mean (SD)	Cases(N=21 043) %	Mean (SD)	Controls (N=105 215) %	Mean (SD)
Socioeconomic status								
Academics	4.9		4.8		4.7		4.7	
Middle education	9.7		8.9		11.7		10.1	
Shorter education	15.5		14.7		19.2		18.9	
Skilled	20.9		21.2		26.3		26.9	
Unskilled	18.5		19.1		22.7		24.5	
Unknown	30.5		31.3		15.4		14.9	
Reproductive factors								
Age (years) at first live birth ^a^		26.1 (4.9)		25.8 (4.8)		24.7 (4.7)		24.5 (4.5)
<25	43.9		47.7		57.7		59.9	
25-29	34.7		33.6		28.3		28.2	
30-34	16.5		13.9		10.2		8.8	
≥35	4.9		4.8		3.8		3.1	
Parity		1.8 (1.2)		1.9 (1.0)		1.9 (1.1)		1.9 (1.3)
0	13.5		11.6		10.7		10.3	
1-2	82.7		83.0		84.6		84.2	
≥3	3.8		5.4		4.7		5.5	
Work-related physical activity								
Ever	48.6		47.9		52.5		51.9	
Never	51.4		52.1		47.5		48.1	
Breast cancer subtypes								
Estrogen receptor negative	23.2				14.5			
Estrogen receptor positive	60.6				74.1			
Missing	16.2				11.4			

a Among parous women.

Adjustment for the selected potential confounders did not affect our results, which showed no striking elevation in the risk of breast cancer before or after age 50 years in women exposed to diesel exhaust and PAH (table 2). Cumulative exposure to diesel exhaust tended to increase the risk before age 50 in a dose–response-like pattern. However, analyses by other dimensions of exposure as well as exposure time windows did not indicate any convincing positive associations for diesel exhaust and PAH in both age groups (table 3). Parous status was shown to affect the association between diesel exhaust and overall breast cancer after age 50 years as only exposed nulliparous women in this age group had an increased risk (OR 1.61, 95% CI 1.13–2.31) (table 4).

**Table 2:** Observed number of cases, controls and odds ratios (OR) with 95% confidence intervals (CI) for breast cancer before and after age 50 among Danish women with diesel exhaust and polycyclic aromatic hydrocarbons (PAH) exposure.

Agent	Total population of women						Subgroup of women ^a^					
	
	Cases	Controls	OR	95% CI	OR ^b^	95% CI	Cases	Controls	OR	95% CI	OR ^c^	95% CI
<50 years												
Diesel exhaust	999	4616	1.08	1.01–1.16	1.07	1.00–1.15	720	3176	1.15	1.06–1.26	1.14	1.05–1.25
PAH	912	4719	0.96	0.89–1.03	0.95	0.88–1.02	631	3208	0.95	0.87–1.05	0.94	0.85–1.03
≥50 years												
Diesel exhaust	1294	6163	1.05	0.98–1.12	1.04	0.98–1.11	1105	5273	1.05	0.98–1.13	1.05	0.98–1.13
PAH	1304	6420	1.01	0.95–1.08	1.00	0.94–1.07	1104	5439	1.05	0.95–1.09	1.01	0.94–1.09

a Women with information on socioeconomic status (SES).

b Adjusted for parity, age at first live birth and work-related physical activity.

c Adjusted for SES.

**Table 3:** Observed number of exposed cases (obs.) and odds ratios (OR) with 95% confidence intervals (CI) for breast cancer before and after age 50 among Danish women with diesel exhaust and polycyclic aromatic hydrocarbons (PAH) exposure by various dimensions of exposure and latency.

	<50 years								≥50 years							
	
	Diesel exhaust				PAH				Diesel exhaust				PAH			
			
	Obs.	OR ^a^	95% CI	P-value	Obs.	OR ^a^	95% CI	P-value	Obs.	OR ^a^	95% CI	P-value	Obs.	OR ^a^	95% CI	P-value
Exposure duration (years)																
1–9	812	1.05	0.97–1.13		739	0.95	0.87–1.03		1045	1.10	1.03–1.18		972	1.02	0.95–1.09	
10–20	146	1.27	1.06–1.52		130	0.97	0.80–1.17		153	0.80	0.67–0.95		199	1.01	0.87–1.18	
>20 years	41	1.04	0.74–1.47		43	0.86	0.62–1.20		97	0.94	0.75–1.16		133	0.91	0.75–1.10	
Trend test				0.00				0.30				0.12				0.98
Cumulative exposure (%) ^b^																
>0–25	212	0.98	0.84–1.13		150	0.88	0.74–1.05		322	1.13	1.00–1.28		239	0.94	0.82–1.08	
>25–50	245	1.01	0.88–1.16		169	0.91	0.77–1.08		332	1.03	0.91–1.16		403	1.05	0.94–1.18	
>50–75	254	1.11	0.96–1.27		345	1.03	0.91–1.16		328	1.02	0.90–1.15		346	1.05	0.93–1.18	
>75	288	1.19	1.05–1.36		248	0.92	0.80–1.05		312	0.99	0.88–1.12		316	0.95	0.84–1.07	
Trend test				0.00				0.48				0.35				0.68
Latency (years) ^c^																
1–9	209	1.07	0.92–1.24		205	1.01	0.87–1.18		128	1.00	0.83–1.22		98	0.90	0.76–1.19	
10–20	355	1.02	0.91–1.15		333	0.97	0.86–1.09		200	1.01	0.87–1.18		209	1.13	0.94–1.29	
>20	435	1.12	1.01–1.25		374	0.90	0.81–1.01		966	1.05	0.98–1.13		997	0.99	0.92–1.08	
Timing of first exposure ^d^																
Prior to first live birth	509	1.06	0.95–1.17		441	0.93	0.83–1.03		471	1.06	0.95–1.17		492	0.96	0.87–1.06	
After first live birth	349	1.07	0.95–1.21		368	1.03	0.92–1.16		639	0.97	0.88–1.06		689	1.06	0.97–1.15	

a Adjusted for parity, age at first live birth and work-related physical activity.

b Probability×intensity×years summed over all exposed time periods in all exposed jobs and categorized according to the percentiles among the controls.

c Years between first exposure and diagnosis.

d Among parous women.

**Table 4:** Observed number of exposed cases (obs.) in Danish women and odds ratios (OR) with 95% confidence intervals (CI) by parous status and age at diagnosis.

Agent	Parous			Nulliparous		
	
	Obs.	OR ^a^	95% CI	Obs.	OR ^b^	95% CI
Diesel exhaust						
<50 years	858	1.06	0.98–1.15	141	1.08	0.77–1.52
≥50 years	1110	1.00	0.94–1.07	184	1.61	1.13–2.31
PAH						
<50 years	809	0.97	0.90–1.05	103	0.57	0.38–0.86
≥50 years	1181	1.02	0.95–1.09	123	1.19	0.80–1.75

a Adjusted for parity, age at first live birth and work-related physical activity.

b Work-related physical activity.

When conducting a further stratification by hormonal subtype of breast cancer, diesel exhaust was primarily associated with a modest increased risk of ER- tumors before age 50 (OR 1.26, 95% CI 1.09–1.46). Before age 50, longer duration of exposure to diesel exhaust was also positively associated with the risk of ER- tumors and cumulative exposure was indicated to increase the risk of both ER- and ER+ breast cancers in a dose–response-like manner, but this observation was most pronounced for ER- tumors. In addition, women exposed >20 years prior to the index date had the highest risk of ER- tumors (OR 1.49, 95% CI 1.19–1.88). Exposure to diesel exhaust after first live birth also increased the risk of ER- breast cancer before age 50 (OR 1.39, 95% CI 1.09–1.76). After age 50, no convincing associations between diesel exhaust and hormonal subtypes of breast cancer were observed (table 5). Subsequent adjustment for PAH exposure did not alter these findings (data not shown). No substantial risk patterns were generally observed for PAH exposure and hormonal subtypes in both age groups (supplementary table S2), and adjustment for diesel exhaust did not affect these findings either (data not shown). Excluding women exposed after age 50 did not affect the risk estimates.

**Table 5:** Observed number of exposed cases (obs.) among Danish women (N=198 888) and odds ratios (OR) with 95% confidence intervals (CI) by age group and estrogen receptor (ER) status, and various dimensions and time windows of exposure relating to diesel exhaust.

	<50 years								≥50 years							
	
	ER-				ER+				ER-				ER+			
			
Obs.	OR ^a^	95% CI	P-value	Obs.	OR ^a^	95% CI	P-value	Obs.	OR ^a^	95% CI	P-value	Obs.	OR ^a^	95% CI	P-value
Overall exposure	243	1.26	1.09–1.46		634	1.05	0.96–1.15		203	1.16	0.99–1.36		971	1.07	1.00–1.15	
Exposure duration (years)																
1–9	202	1.25	1.07–1.47		508	1.02	0.92–1.13		171	1.26	1.06–1.50		779	1.13	1.04–1.23	
10–20	30	1.20	0.80–1.80		97	1.27	1.01–1.59		20	0.77	0.47–1.21		118	0.83	0.68–1.01	
>20	11	1.60	0.81–3.15		29	1.00	0.67–1.50		12	0.89	0.48–1.65		74	0.97	0.75–1.25	
Trend test				<0.00				0.07				0.52				0.34
Cumulative exposure (%) ^b^																
>0–25	50	1.12	0.82–1.52		138	0.95	0.79–1.14		62	1.53	1.15–2.04		238	1.14	0.99–1.32	
>25–50	60	1.21	0.90–1.61		141	1.00	0.83–1.20		48	1.07	0.78–1.46		251	1.08	0.94–1.24	
>50–75	65	1.34	1.01–1.77		173	1.06	0.90–1.25		54	1.18	0.87–1.59		242	1.03	0.90–1.19	
>75	68	1.36	1.04–1.79		182	1.18	1.00–1.39		39	0.88	0.62–1.24		240	1.04	0.90–1.20	
Trend test (P–value)				<0.00				0.02				0.58				0.09
Latency (years) ^c^																
<10	58	1.33	0.99–1.79		115	1.00	0.82–1.22		20	1.06	0.65–1.72		97	1.06	0.85–1.32	
10–20	85	1.03	0.81–1.31		217	1.04	0.90–1.21		34	1.15	0.79–1.68		145	1.02	0.85–1.22	
>20	100	1.49	1.19–1.88		302	1.08	0.95–1.22		149	1.17	0.97–1.41		729	1.09	1.00–1.18	
Timing of exposure ^d^																
Before first live birth	110	1.11	0.89–1.37		341	1.07	0.95–1.22		63	1.01	0.76–1.34		364	1.12	1.00–1.26	
After first live birth	94	1.39	1.09–1.76		210	1.00	0.86–1.16		110	1.10	0.89–1.36		472	0.98	0.89–1.09	

a Adjusted for parity, age at first live birth and work-related physical activity.

b Probability*intensity*years summed over all exposed time periods in all exposed jobs and categorized according to the percentiles among the controls.

c Years between first exposure and diagnosis.

d Among parous women.

## Discussion

The present study showed no marked elevated risk of overall breast cancer diagnosed before or after age 50 following occupational exposure to diesel exhaust and PAH, respectively. However, diesel exhaust modestly elevated the risk of early-onset (before age 50) ER- tumors, and duration– and dose–response relationships were also observed for this particular subtype in this age group as well as a positive association with longer latency. Diesel exhaust exposure after first live birth increased the risk of early-onset ER- tumors. After age 50, diesel exhaust was associated with an increased risk of overall breast cancer among nulliparous women. No other notable positive risk patterns were observed for diesel exhaust exposure after age 50 as well as for PAH exposure in both age groups.

Previous similar studies exploring the association between diesel exposure and breast cancer incidence in women are somewhat inconsistent. A Swedish cohort study using a similar JEM for the exposure assessment reported an increased risk of post-menopausal breast cancer with longer duration (HR 1.69, 95% CI 1.01–2.82) and higher cumulative exposure (HR 1.61, 95% CI 0.93–2.79) (17). Using the same cohort, a subsequent study with improved exposure estimates also detected an increased risk of post-menopausal breast cancer among women with diesel exhaust exposure (22). These findings do not resemble those observed in this present study, which only indicated an increased risk of early-onset ER- tumors. The Swedish study population was somewhat smaller compared to ours and a different categorization of employment was used, which may in part explain the inconsistency in results.

An Australian case–control study used occupational experts to assess exposure to diesel exhaust and reported a modest increased risk of pre-menopausal breast cancer (OR 1.29, 95% CI 0.77–2.18), but no risk elevations were observed for post-menopausal breast cancer (20). A Finish case-control study also using a JEM for the exposure assessment similarly detected an increased risk of pre-menopausal breast cancer in women with medium/high level of exposure to diesel exhaust (SIR 1.48, 95% CI 0.48–4.61) (15) while no association between occupational exposure to diesel exhaust and overall breast cancer was detected in a Swedish cohort study (18). These last reports in part support our results as we did not detect an overall increased risk of breast cancer before or after age 50 and, moreover, only observed an indication of an increased risk of early-onset breast cancer with cumulative exposure. However, when stratifying by ER status, we observed a somewhat consistent pattern of an increased risk of ER- tumors before age 50. As these previous studies did not stratify by ER status in women with pre-menopausal breast cancer, a potential increased risk of early-onset ER- tumors may have been overlooked.

Our somewhat consistent findings of an increased risk of ER- tumors before age 50 among women exposed to diesel exhaust may be biologically plausible; diesel exhaust is classified as carcinogenic to humans (8) and chemical components are lipophilic, have estrogenic properties, and are stored in mammary tissue where they may cause carcinogenesis (10). Further, it has been theorized that ER- tumors may be more sensitive to hormonal imbalance and that they are therefore more strongly affected by acknowledged risk factors (31). Hence, it is possible that diesel exhaust may affect the risk of this subtype more strongly as well. Moreover, young women exposed to diesel exhaust may have an increased breast cancer risk as they experience several time windows of heightened biological susceptibility to carcinogenic exposures, ie, the time prior to first full-term pregnancy, where breast cells are less differentiated, and during pregnancy, where hormones and growth factors mediate maximal development of breast tissue (32). However, only exposure to diesel exhaust after first live birth was observed to increase the risk of early-onset ER- tumors in our study, which implies that the time after pregnancy may also constitute a window of susceptibility to carcinogenic exposures increasing the risk of this breast cancer subtype. However, this observation cannot be confirmed by previous studies as similar examinations have not been undertaken. Since the period following birth has not been highlighted as critical in a recent review on influences of environmental chemicals on breast cancer risk (11), our observation may as well be due to chance or uncontrolled confounding and therefore needs to be studied further.

In this present study, nulliparous women exposed to diesel exhaust were observed to have an increased risk after age 50, which supports the hypotheses that undifferentiated breast cell structures in these women are more susceptible to carcinogenic exposures (12–14). To the authors’ knowledge, no prior incidence studies have addressed the risk of breast cancer among nulliparous women with occupational diesel exhaust exposure as well. However, a previous study on breast cancer risk with occupational exposure to benzene, which is one of the chemical components in diesel exhaust, reported an elevated risk among nulliparous women (OR 1.94, 95% CI 0.9–4.1) (33) and thus partly supports our finding. However, this result needs to be confirmed in future studies.

Our explorations of a potential effect of PAH exposure on breast cancer risk did not yield any convincing positive findings. This is generally not supported by previous incidence studies indicating an increased breast cancer risk among women with occupational PAH exposure (16, 17, 19, 21). When using a JEM for the exposure assessment, all workers employed within the same industry are assigned the same exposure, despite the fact that there may well be exposure variance due to factors such as job, job tasks and protective equipment. In addition, we used a JEM that was not gender-specific, which may also be considered a limitation as women and men in the same industry may be exposed differently due to different jobs and job tasks. Consequently, non-differential exposure misclassification may have been an issue and led to an attenuation of risk estimates. However, as we considered women in exposed jobs with a probability of <20% to be unexposed, exposure misclassification may have been reduced. In addition, the JEM entailed specific dimensions such as specific time periods, probability and intensity, which are features that have been shown to increase validity and reduce the attenuation of risk estimates (34). As several prior studies detecting an increased breast cancer risk with PAH exposure also used a JEM with no gender-specific dimensions to assess exposure to PAH (17, 19, 21), additional methodological issues may as well have contributed to the discrepancy in results. Other limitations in our study involve that the oldest generation of women included in the study that held jobs in a very young age, ie, <18 years, might have been misclassified with respect to exposure as these jobs would not have been registered in the ATP register. Further, the classification of employment in the various versions of the NOCCA-JEM was based on different classifications of employment. Hence, the crosswalk between the original NOCCA-JEM and the Danish version was somewhat imperfect and may have increased the exposure misclassification slightly. Moreover, we did not have information on exposure after 1995 and, therefore, metrics of probability and level of exposure in this period may have been slightly imprecise as they were based on metrics in the most recent era in the JEM (1985–1995). As the ATP register has high validity (26), other misclassification errors regarding industrial classification were not considered to have weakened our results. The Danish Cancer Registry and DBCG also have high validity and almost complete coverage in the study period (23) (24), and therefore we do not consider misclassification of breast cancer status to be an issue either.

More importantly, it was not possible to account for potential confounding due to certain lifestyle factors such as obesity, alcohol consumption, physical inactivity, use of oral contraceptives and hormone replacement therapy (4). As several of these factors are associated with socioeconomic group, we may have indirectly accounted for these potential effects by controlling for the variable SES in our full case analyses, which did not change our risk estimates. These analyses may still be considered somewhat limited since our SES variable was based on self-reported job title and a relatively high percentage of the women in our study had missing information on this variable. However, as most of these lifestyle factors present a modest risk of breast cancer (35) and adjustment for these variables in most previous studies in this research area had marginal or no effect on the diesel exhaust and PAH exposure risk estimates (16, 17, 20, 21), lack of this information in our study are not presumed to be a critical limitation. Nonetheless, as our overall risk estimates are considered somewhat modest, they may still be explained by unknown confounding or chance. We choose not to make adjustment for multiple comparisons, as it has been argued that this strategy will lead to fewer errors of interpretation (36). Instead, positive associations were discussed according to biological plausibility and compared with the existing literature.

The exposures under study are also found in the general environment, however, normally to a lower extent than in some sectors of the working environment. Not being able to account for individual level exposure outside work is generally considered to be a limitation in occupational studies, including this one.

The strengths of this study include the large nationwide population-based case–control design, which allowed us to evaluate the risk of a high number of incident breast cancer cases, including specific subtypes, with life-time occupational exposures. Moreover, a uniqueness of the study was its unusual high number of breast cancer cases in relatively young women allowing us to explore rare subtypes by different age groups. Using reliable registry data on occupational history and a validated JEM with objective workplace exposure measurements and features such as probability and intensity scores in different time periods, which refined the exposure assessment, were also considered major strengths. Especially information on lifetime occupational history on Danish women with historical high workforce participation rates (37) allowed us not only to examine breast cancer, by ever having worked in an exposed job, but also by various exposure measures.

### Concluding remarks

This study shows no notable association between occupational diesel exhaust and overall breast cancer risk, and the same applies to PAH exposure. However, our results show a pattern indicating that diesel exhaust may increase the risk of ER- tumors in women before 50 years. Future studies on this issue that differentiate between subtypes of breast cancer in different age groups and explore the effect of reproductive status and exposure time windows are needed.

## Supplementary material

Supplementary material
