# Supplementary material for: Breast cancer among Danish women occupationally exposed to diesel exhaust and polycyclic aromatic hydrocarbons, 1964–2016
Source: Scand J Work Environ Health. 2021 Mar 1;47(2):154–62. doi: 10.5271/sjweh.3923 (PMC8114566; doi:10.5271/sjweh.3923)

Breast cancer among Danish women occupationally exposed to diesel exhaust and polycyclic aromatic hydrocarbons, 1964-2016[1]

by Julie Elbæk Pedersen, MSc,[2] Katrine Strandberg-Larsen, PhD, Michael Andersson, PhD, Johnni Hansen, PhD

1. *Supplementary material*
2. *Correspondence to: Julie Elbæk Pedersen, Danish Cancer Society Research Center, Strandboulevarden 49, 2100 Copenhagen Ø, Denmark. [E-mail: juliep@cancer.dk]*

Supplementary table 1. Danish industries with corresponding DSE codes and proportion of exposed women in the study population ever employed in specific industries

Industry	DSE	%
Diesel exhaust		
Construction	50121	54,1
Shipping companies	71211	42,0
Carrier businesses	71141	24,8
Railroads	71110	20,9
Other transportation businesses	71149	14,0
Bus operation	71121	11,3
Taxi drivers	71132	10,6
Service stations	62213	9,5
Building and carpentry firms	50140	8,2
Sightseeing bus drivers	71131	7,5
Ferry operations	71212	5,3
Asphalt factories	35401	3,0
Taxi dispatchers	71133	2,2
Moving businesses	71142	1,7
Harbours	71232	1,1
Loading and unloading contractors (stevedores), etc.	71233	0,9

Freight depots	71143	0,8
Other building activities	50199	0,7
Fire brigade	71163	0,5
Construction of roads, tunnels, railways, etc.	50125	0,0
Total proportion of exposed women in the study population: 7%		
PAHs		
Manufacture of other machinery	38299	30,9
Manufacture of iron and metal structures	38131	17,7
Iron shipyards	38411	13,4
Manufactures of office machinery	38251	11,5
Manufacture of other household equipment	38293	11,2
Manufacture of agricultural machinery and accessories	38220	10,7
Manufacture of machinery and equipment for food industry	38242	10,7
Forge- and machine repair workshops	38280	9,6
Manufacture of other industrial machinery	38249	8,9
Iron foundries	37102	7,6
Manufacture of internal transport equipment	38294	7,6
Subcontracting factories manufacturing iron and metal	38298	4,1
Metal foundries	37202	3,5
Manufacture of stationary tanks	38132	3,3
Manufacture of machinery for metalworking	38232	3,2
Asphalt factories	35401	3,0
Marine engine manufacture	38413	2,8
Manufacture of stoves and cookers for household use	38292	2,6
Metal works	37201	2,1

Manufacture of machinery for woodworking	38231	1,6
Manufacture of automobiles	38431	1,4
Loading and unloading contractors (stevedores), etc.	71233	0,9
Manufacture of textile machinery and accessories	38241	0,7
Manufacture of machinery for shoe-making, paper and cardboards	38243	0,7
Manufacture and repair of sewing machines	38295	0,7
Manufacture of gearwheels and transmissions	38296	0,6
Manufacture of engines (except for electric motors)	38210	0,6
Chimney sweepers	92023	0,5
Manufacture of institutional kitchen equipment	38291	0,5
Manufactures of scales	38252	0,5
Manufacture of machinery for dry-cleaning establishments	38297	0,5
Manufacture of foundry machinery	38233	0,1
Construction of roads, tunnels, railways, etc.	50125	0,0
Total proportion of exposed women in the study population: 6%		

Supplementary table 2. Observed number of exposed cases (obs.) in Danish women (N=198,888) and odds ratios (ORs) with 95% confidence intervals
(95% CI) by age group and estrogen receptor (ER) status and various dimensions and time windows of exposure to polycyclic aromatic hydrocarbons (PAHs)

	<50 years						≥50 years					
	ER-			ER+			ER-			ER+		
	Obs.	OR[a]	95% CI	Obs.	OR[a]	95% CI	Obs.	OR[a]	95% CI	Obs.	OR[a]	95% CI
Overall exposure	216	0.99	0.85-1.16	579	0.95	0.86-1.04	187	0.99	0.84-1.18	977	1.02	0.95-1.10

Duration of exposure												
1-9 years	168	0.98	0.82-1.17	468	0.95	0.85-1.05	135	0.96	0.79-1.17	727	1.04	0.95-1.13
10-20 years	37	1.05	0.73-1.51	83	0.99	0.78-1.26	32	1.25	0.84-1.86	148	1.01	0.85-1.21
>20 years	11	0.96	0.50-1.84	28	0.88	0.58-1.32	20	0.91	0.56-1.48	102	0.94	0.76-1.17
Trend test (P-value)	0.92			0.56			0.95			0.56		
Cumulative exposure[b]												
>0-25%	44	1.11	0.80-1.55	90	0.80	0.63-1.00	37	0.89	0.62-1.28	182	0.97	0.82-1.14
>25-50%	38	0.87	0.61-1.24	111	0.93	0.75-1.14	55	1.11	0.83-1.50	288	1.02	0.90-1.16
>50-75%	69	0.96	0.73-1.25	227	1.07	0.92-1.24	49	1.02	0.74-1.39	267	1.12	0.98-1.29
>75%	65	1.03	0.79-1.36	151	0.92	0.77-1.10	46	0.93	0.68-1.29	240	0.97	0.80-1.12
Trend test (P-value)	0.99			0.90			0.83			0.28		
Latency[c]												
<10 years	55	1.11	0.82-1.50	117	1.00	0.82-1.23	17	1.08	0.63-1.82	67	0.81	0.62-1.06
10-20 years	83	1.07	0.84-1.37	197	0.93	0.79-1.09	30	1.15	0.77-1.72	159	1.15	0.97-1.37
>20 years	78	0.86	0.67-1.10	265	0.95	0.83-1.09	140	0.96	0.79-1.16	751	1.02	0.94-1.11
Timing of exposure[d]												
Before first live birth	106	1.02	0.82-1.27	286	0.93	0.82-1.07	72	0.98	0.76-1.28	364	0.96	0.86-1.09

After first live birth	86	1.10	0.86-1.40	233	1.04	0.90-1.21	97	1.01	0.80-1.27	519	1.07	0.97-1.19

[a]Adjusted for parity, age at first live birth and work-related physical activity
[b]Probability*intensity*years summed over all exposed time periods in all exposed jobs and categorized according to the percentiles among the controls
[c]Years between first exposure and diagnosis
[d]Among parous women

Supplementary figure 1. Directed Acyclic Graph (DAG) for confounder selection in the association between diesel exhaust and PAH exposure and breast cancer risk.

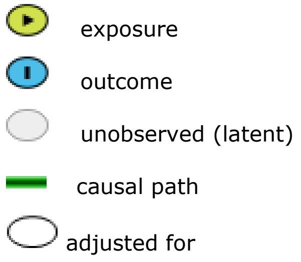

Supplement: Supplementary material [file SJWEH-47-154-S001.pdf]
